# Antimicrobial Activity of Zinc Oxide Nano/Microparticles and Their Combinations against Pathogenic Microorganisms for Biomedical Applications: From Physicochemical Characteristics to Pharmacological Aspects

**DOI:** 10.3390/nano11020263

**Published:** 2021-01-20

**Authors:** Su-Eon Jin, Hyo-Eon Jin

**Affiliations:** 1 Research Institute for Medical Sciences, College of Medicine, Inha University, Incheon 22212, Korea; 2 College of Pharmacy, Ajou University, Suwon 16499, Korea

**Keywords:** zinc oxide nano/microparticles, antimicrobial activity, nanoantibiotics, physicochemical characteristics, biomedical application

## Abstract

Zinc oxide (ZnO) nano/microparticles (NPs/MPs) have been studied as antibiotics to enhance antimicrobial activity against pathogenic bacteria and viruses with or without antibiotic resistance. They have unique physicochemical characteristics that can affect biological and toxicological responses in microorganisms. Metal ion release, particle adsorption, and reactive oxygen species generation are the main mechanisms underlying their antimicrobial action. In this review, we describe the physicochemical characteristics of ZnO NPs/MPs related to biological and toxicological effects and discuss the recent findings of the antimicrobial activity of ZnO NPs/MPs and their combinations with other materials against pathogenic microorganisms. Current biomedical applications of ZnO NPs/MPs and combinations with other materials are also presented. This review will provide the better understanding of ZnO NPs/MPs as antibiotic alternatives and aid in further development of antibiotic agents for industrial and clinical applications.

## 1. Introduction

Zinc oxide (ZnO) nanoparticles (NPs) have been studied for the development of next-generation nanoantibiotics against pathogenic microorganisms to combat multi-drug resistance [1,2]. These nanoparticles show unique physicochemical properties including morphology, particle size, crystallinity, and porosity [3]. Based on these characteristics, ZnO NPs have a wide spectrum of antimicrobial activity against microorganisms including *Escherichia coli*, *Staphylococcus aureus*, *Pseudomonas aeruginosa*, *Bacillus subtilis*, and the M13 bacteriophage [4,5,6,7]. They can be combined with antibiotic and anti-inflammatory drugs to enhance antimicrobial activity against pathogenic microorganisms without antibiotic resistance in non-clinical and clinical conditions [7,8]. Use of ZnO NPs as well as microparticles (MPs) has been extended to biomedical applications including antibiotic drugs or medical devices, theranostics, implants, and cosmetics for conventional uses in clinics [9,10,11,12].

The physicochemical characterization of ZnO NPs/MPs offers advantageous information regarding biological or biochemical responses to pathogenic microorganisms, enabling the prediction of antimicrobial and toxicological effects [13,14]. Generally, their properties, including morphology, particle size or particle size distribution, porosity, and specific surface area, can affect antimicrobial responses [14,15]. ZnO NPs/MPs of various shapes such as spheres, rods, needles, and platelets, and their average aspect ratios (defined as ratio of length to width) can influence the antimicrobial actions against microorganisms. Their particle size and particle size distribution levels are the key parameters that determine NP uptake into biomembranes and thereby influence antimicrobial activity against pathogenic microorganisms [16,17]. Intra- and interparticle pores of ZnO NPs/MPs also enhance photocatalytic antimicrobial interactions under ultraviolet (UV) and visible light irradiation based on reactive oxygen species (ROS) [5,18]. Furthermore, large specific surface area levels of ZnO NPs/MPs facilitate membrane adsorption for antimicrobial actions [19].

Furthermore, combinations of ZnO NPs/MPs with other antibiotic drugs, metal oxide NPs/MPs, and devices have been used to enhance antimicrobial activity against pathogenic microorganisms [20,21,22,23,24]. They have synergistic or improved antimicrobial activity against *E. coli*, *S. aureus*, *Aeromonas veronii*, *P. aeruginosa*, *B. subtilis*, and *Klebsiella pneumoniae*. ZnO NP/MP combinations with other materials are applied as dosage forms other than particles, including membranes, films, and plates, according to the administration route or usage for enhanced antimicrobial performance [25,26,27,28]. These dosage forms can deliver the drug to the specific site of infection in human diseases including endocarditis, cystic fibrosis, pneumonia, and otitis [29,30].

In this review, we introduce the physicochemical characteristics of ZnO NPs/MPs to explain their antimicrobial activity against pathogenic microorganisms. The antimicrobial activity of ZnO NPs/MPs and their combinations with other antimicrobial materials such as antibiotic and anti-inflammatory drugs, metal oxide NPs/MPs, and polymers is also described. Moreover, current biomedical applications of ZnO NPs/MPs are presented.

## 2. Characteristics of ZnO Materials Based on Synthesis Techniques

ZnO NPs (81.38 g/mol, <100 nm) are white odorless solid powders of hexagonal wurtzite crystals with various shapes including spheres for zero-dimensional (0D) structures, dumbbells, nanorods, nanotubes, and needles for one-dimensional (1D) structures, disks and platelets for two-dimensional (2D) structures, and flowers, stars and flakes for three-dimensional (3D) structures [6]. They have a wide band gap energy (~3.3 eV) which is similar to that of titanium dioxide (TiO_2_) NPs. Compared to ZnO NPs, ZnO MPs are generally in the submicron range regarding their size.

The physicochemical properties of ZnO materials mainly affect their antimicrobial activity against pathogenic microorganisms and are critical parameters linked to pharmacological and toxicological responses [31,32]. Their morphology, particle size, and porosity are determined to confer the superior antimicrobial activity in pathogenic microorganisms [3]. These physicochemical properties of ZnO NPs/MPs are influenced by the synthesis techniques used for their preparation [5,6,33].

### 2.1. Synthesis Techniques of ZnO Materials

The synthesis techniques used for ZnO NPs/MPs are generally categorized by physical, chemical, biological, and microfluidic methods [5,34]. Physical methods of arc plasma, thermal evaporation, physical vapor deposition, ultrasonic irradiation, and laser ablation simply produce chemically pure ZnO NPs/MPs. Wet chemical methods for the synthesis of ZnO NPs/MPs such as microemulsion, sol–gel, precipitation, hydrothermal and solvothermal methods are extensively used to generate the specific physicochemical characteristics, based on their simple and scalable bottom-up approaches [35,36]. Biological methods, so called “green synthesis”, are promoted as ecofriendly synthesis techniques including microorganisms (bacteria, fungi, yeasts, algae, and phages), plant extracts, DNA, and proteins [5,37]. The biosynthesized ZnO NPs/MPs have comparable physicochemical characteristics to those of physically or chemically synthesized ZnO NPs/MPs. Moreover, microfluidic methods can offer high-value ZnO NP/MP products using modular architecture integration for sophisticated reactions [5,38], which enable ZnO NPs/MPs to have targeted physicochemical properties.

Meanwhile, various electrochemical processes for synthesis of ZnO NPs/MPs have been reported including electrodeposition [39,40], sacrificial anode electrolysis [41], and electrochemical deposition under oxidizing conditions (EDOC) [42,43]. Hybrid techniques of electrochemical-thermal processes were also reported in aqueous environment [44,45]. In electrodeposition, ZnO thin films can be produced controlling current density, applied potential, time, and electrolytic bath concentration as major operating parameters using zinc nitrate solution or zinc chloride solution as precursors [40]. This technique is advantageous to achieve simple and cost-effective outcomes in large-surfaced substrate, which produces various structures of nanoneedle-like, prism-like, porous, and continuous shapes with appropriate thickness [39]. On silicon, ZnO deposition occurred from nonaqueous solution of dimethyl sulfoxide (DMSO) containing zinc chloride and potassium chloride, which films of 1–3 μm thick had pore channels (20 nm). Next, sacrificial anode electrolysis from two-electrode cells to thee-electrode cells along with potentiostatic control improves reproducibility of morphological dimension in ZnO NPs/MPs [41]. In the electrode-cell, metal ions are produced in anodic dissolution or added into electrochemical medium and cathodic reduction induces particle growth with tunable morphology. Near-spherical ZnO NPs (<25 nm) in an amorphous phase were produced using sacrificial anodic dissolution of pure zinc metal strip (2 × 6 × 1 cm^3^) controlling against platinum mesh as the cathode in electrolyte of 0.1 M tetrabutlyammonium bromide solution and acetonitrile (4:1) [46]. In EDOC, sacrificial anodic oxidation, cathodic reduction, and oxidation processes were involved for the synthesis of spherical ZnO NPs (9 nm) [42]. Hybridizing electrochemical and thermal approaches, ZnO NPs/MPs were synthesized in aqueous sodium bicarbonate solution, generating zinc hydroxide species and further oxidized to ZnO in calcination process at >300 °C [44,45]. Specifically, anionic or cationic stabilizers can be used to tune physicochemical properties of ZnO NPs/MPs including morphology and particle size.

### 2.2. Physicochemical Characteristics of ZnO Materials

We introduce the physicochemical characteristics of ZnO NPs/MPs which antimicrobial functions have already been reported. Table 1 displays morphology, particle size, porosity, surface area, and synthesis technique of ZnO NPs/MPs with antimicrobial functions against pathogenic microorganisms. In general, high aspect ratio, small particle size, multilevel porosity, and large surface area of ZnO NPs/MPs can enhance antimicrobial performance, irrespective of synthesis techniques [3,5,14]. Chemically or biologically synthesized ZnO NPs/MPs have been extensively used to determine their antimicrobial activity against microorganisms, rather than microfluidically or electrochemically synthesized ZnO NPs/MPs, due to simplicity, reproducibility, and production scale, yield or rate for obtaining high-value ZnO NPs/MPs with required characteristics [5,6,8].

First, in the 0D structure of ZnO NPs/MPs, Raghupathi et al. [16] reported the manufacture of spherical ZnO NPs (12–307 nm) with slit-like pores (3.49–90.4 m^2^/g surface area) using solvothermal and room temperature syntheses. Using the solvothermal technique for ZnO NP synthesis, smaller ZnO NPs (12–25 nm) with larger surface area (42.8–90.4 m^2^/g) were obtained, compared to ZnO NP synthesis at room temperature (30–307 nm; 3.49–35.6 m^2^/g surface area). Green synthesized spherical ZnO NPs were reported, using *Catharanthus roseus* leaf extract and zinc acetate dehydrate [47]. In spherical ZnO NP synthesis, controllable reaction factors were optimized for pH 12, 30 °C, 0.01 M precursor metal ion, and 2 h of reaction time to achieve high-value ZnO NPs for enhanced antimicrobial performance. Using green synthesis and co-precipitation, sphere-shaped ZnO NPs (15–20 nm) were obtained reacting with *Bambusa Vulgaris* plant extract and zinc nitrate aqueous solution (0.5 mol/50 mL) [48]. Compared to *Bambusa Vularis* plant extract, *Artabotrys Hexapetalu* plant extract was used to synthesize mixed spherical and rod-like ZnO NPs (20–30 nm). In green synthesis using *Sambucus ebulus* leaf extract and 1 M zinc acetate dihydrate, spherical ZnO NPs were also prepared at 25–30 nm for transmission electron microscopy (TEM) and 65 ± 4 nm for dynamic light scattering [49]. Moreover, a self-assembled 3D network structure of spherical ZnO NPs (48.3 nm) on a solid plate was also analyzed, in which NPs were synthesized via sol–gel technique using an annealing process at 150 °C [18]. In the self-assembled ZnO NP network, mesopores at 5–6.25 nm and macropores at 2–6 μm were detected, conferring bimodal porosity for enhanced performance. Meanwhile, spherical nanostructured ZnO (63 nm) manufactured by soft chemical synthesis using micrometric ZnO and urea in glycerol, generated NP clusters (590 nm) with mesopores of 34 nm and a 20.72 m^2^/g surface area [19].

In the 1D structure of ZnO NPs/MPs, Bala et al. [50] reported dumbbell-shaped ZnO NPs (190–250 nm in length and 50–60 nm in breadth), which were prepared via green synthesis by reacting with *Hibiscus subdariffa* leaf extract and zinc acetate in water. The morphology of ZnO NPs was modified depending on drying temperature. After particle precipitation, dumbbell-shaped ZnO NPs were generated with further heating at 100 °C for 4 h. In contrast to the dumbbell-shaped ZnO NPs, sphere-shaped ZnO NPs (12–46 nm) resulted from drying at 60 °C. The submicron ZnO dumbbell structure was generated using a chemical bath deposition technique at low temperature (80 °C) [51]. The resulting ZnO dumbbells were 1–2 μm long, 200–300 nm wide at one end, and 250–400 nm wide at the other end.

ZnO nanorods (523 nm in length and 47 nm in diameter) were reported as photocatalytic antibacterial agents by Singh et al. [52]. They were synthesized using the sol–gel technique by reacting with 1 M zinc nitrate and 1 M hexamethyl tetraamine (HMTA) at pH 6.5. Reactions carried out at pH 5.0–6.5 in mediated smaller ZnO nanorods. Using the green synthesis technique of reacting with egg white albumen and zinc acetate dihydrate in water (1 mmol/mL), the resulting ZnO nanorods had a highly crystalline structure at ~2 μm in length and ~50 nm in diameter for enhanced antimicrobial performance [53]. Calcined temperature was determined as a controllable parameter for high-value ZnO nanorod synthesis, ranged at 400–650 °C. ZnO nanorods calcined at the highest temperature (650 °C) displayed pure and highly crystalline products.

Hollow ZnO nanotubes in nanopowders, ranging at ~500 nm were manufactured via hydrothermal synthesis using polyvinyl alcohol (PVA) with a further annealing process at 500–900 °C [54]. After annealing, the surface area and pore size of the ZnO nanopowders were reduced from 17.8 m^2^/g and 278.6 nm to 2.4–16.7 m^2^/g and 53–255.4 nm, respectively, compared to as-synthesized nanopowders, owing to sintering and growing boundaries to form microscale aggregates. Lopez de Dicastillo et al. [55] described hollow ZnO nanotubes 5 μm in length and 59.5 nm in thickness with an internal diameter of 178.2 nm, prepared via template methods using electrospun PVA nanofibers through atomic layer deposition of ZnO NPs from water and diethyl zinc. Thermal treatment or hydrolysis was additionally performed to remove the PVA template from the hollow ZnO nanotubes. Besides the nanotubes, ZnO needles were also reported as antimicrobial agents for urinary tract pathogens [56]. These needle-shaped ZnO NPs were green synthesized using *Berberis aristata* leaf extract and 0.1 M zinc acetate dihydrate adjusting pH with 1 M sodium hydroxide. Their size range was ~2 μm in length and 20–40 nm in diameter.

In 2D structures of ZnO NPs/MPs, ZnO nanodisks were prepared using co-precipitation by reacting with 1 mM zinc acetate dihydrate and 2 M sodium hydroxide in water at room temperature [57]. The nanodisks, theoretically calculated at 41 nm, showed a minimum toxicity (LD50 for brine shrimps, >200 μg/mL) compared to those synthesized in other solvents, including acetone (size, 29.1 nm; LD50, 83.2 μg/mL), ethyl acetate (size, 40.1 nm; LD50, 140.3 μg/mL), ethanol (size, 33.7 nm; LD50, 90 μg/mL), and methanol (size, 32.8 nm; LD50, 49.4 μg/mL), with the exception of chloroform (size, 38.5 nm; LD50, >200 μg/mL). Platelet-shape ZnO NPs of different sizes (14.7 nm and 17.5 nm) were also reported by Pasquet et al. [58], in which size distribution was less than 100 nm despite the particle size increasing to 5.0–6.4 μm in a hydrodynamic environment. The particles had mesopores of 11.6–17.3 nm and a specific surface area of 39.0–46.9 m^2^/g.

In 3D structured ZnO NPs/MPs such as antibiotic agents, flower-like ZnO MPs were synthesized using electrochemical-thermal method with poly-diallyl-(dimethylammonium) chloride (PDDA) and benzyl-dimethylammonium chloride (BAC) as a stabilizer, which were 200–500 nm or 1–2 μm long [45]. Flower-shaped ZnO NPs were also synthesized using the sol–gel method reacting with 5% zinc acetate dihydrate and 0.2 *w/v*% cetyltrimethyl ammonium bromide (CTAB) at pH 11.7–11.9, and 25–75 °C of near-room temperatures [59]. They showed enhanced antibacterial and antifungal performance via larger zones of growth inhibition when compared to ZnO powders and conventional products. Flower-like ZnO NPs were further reported by Quek et al. [60], which were sized at 700 nm–2.2 μm in diameter for visible light-responsive photocatalytic antimicrobial action. Talebian et al. [61] described higher antibacterial activity of flower-shaped ZnO NPs compared to those of rod- and sphere-shaped ZnO NPs. The flower-shaped ZnO NPs were synthesized using the solvothermal technique reacting with zinc acetate dihydrate in water (5.6 g/50 mL) and 0.5 M sodium hydroxide. They resulted in a smaller particle (45 nm) with larger surface area (28.8 m^2^/g) than ZnO NPs synthesized in 1-hexanol (rod shape; 76 nm; 15.5 m^2^/g surface area) and ethylene glycol (sphere shape; 65 nm; 19.2 m^2^/g surface area), predicting enhanced antimicrobial performance. At this point, it is believed that the solvent in the solvothermal technique is a critical parameter in the synthesis of ZnO NPs, which results in differentiated morphology. Star-shaped ZnO NPs were also synthesized at 31 nm using liquid precipitation for preparing microorganism-protective defense clothing on cotton or polyester fabrics due to their biocompatibility [62]. Using co-precipitation, flake-shaped ZnO NPs were prepared at 32 nm size for enhanced antimicrobial performance [63].

Therefore, irrespective of ZnO NP/MP variety, the physicochemical characteristics of ZnO NPs/MPs affect their antimicrobial activity against pathogenic microorganisms [14,64], and synthesis techniques can be controlled to improve the properties of ZnO NPs/MPs for enhanced antimicrobial performance [13,65].

## 3. Antimicrobial ctivity against Pathogenic Microorganisms

### 3.1. Antimicrobial Mechanisms of ZnO Materials

**Figure 1:**
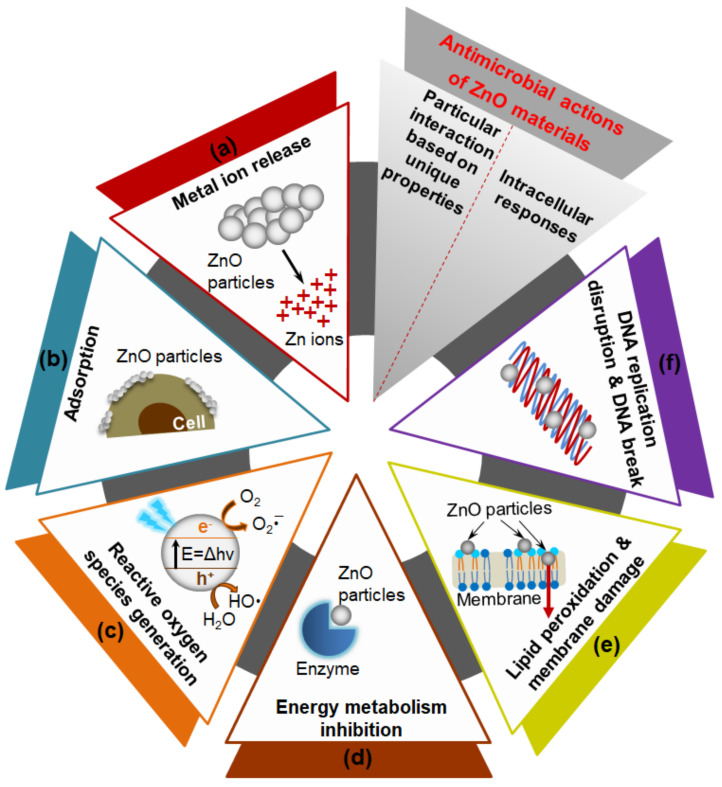
Mechanisms of zinc oxide (ZnO) materials used in antimicrobial applications: Particular interactions of (**a**) metal ion release, (**b**) adsorption, and (**c**) reactive oxygen species generation based on their physicochemical properties; and following intracellular responses of (**d**) energy metabolism inhibition, (**e**) lipid peroxidation and membrane damage, and (**f**) DNA replication disruption and DNA break.

Mechanisms of antimicrobial actions of ZnO materials have been explained in association with particular interaction based on their unique physicochemical properties of (a) Zn^2+^ ion release, (b) adsorption, and (c) ROS generation [14,66], and the intracellular responses in microorganisms of (d) energy metabolism inhibition; (e) lipid peroxidation, and cell membrane damage; and (f) DNA replication disruption, and DNA break [67,68] (Figure 1). The Zn^2+^ ions that are released from ZnO NPs/MPs induce an antimicrobial response in microorganisms due to interference in metabolic processes and disturbance in enzymatic systems [69,70]. ZnO NPs/MPs also have functions of particle adsorption to the biomembrane via a charge–charge interaction, and ROS generation as photocatalysts under UV and visible light irradiation [3,12,71,72]. Positively charged surfaces of ZnO NPs/MPs interact with the negatively charged cell wall or biomembrane of microorganisms [73]. They are internalized into the microorganisms after adsorption resulting in loss of cell integrity based on cell wall or membrane rupture, and further mediate oxidative stress owing to lipid peroxidation leading to deoxyribonucleic acid (DNA) damage. Based on the fundamental mechanisms of action, ZnO NPs/MPs have a differential susceptibility against pathogenic microorganisms, affected by their physicochemical characteristics including morphology, particle size, and porosity [3,17,18,73].

In the photocatalytic activation of ZnO NPs/MPs for ROS generation linked to oxidative stress, electrons (e^−^) are transmitted from the valence band to the conduction band by the photoexcitation over band gap energy (E = Δhν) leaving positive holes (h^+^) [14,65]. Holes in the valence band produce hydroxyl radicals (OH·), whereas electrons in the conduction band generate superoxide radical anions (O_2_·^−^) in an aqueous environment. Reacting with electrons and hydrogens, hydroxyl peroxide radicals (HO_2_·) and finally hydrogen peroxide (H_2_O_2_) are formed. In addition, surface defects of ZnO NPs can enhance ROS production even in the dark for enhanced antimicrobial performance [74].

In 3D structures of ZnO NPs/MPs followed by aggregate formation or nanostructure generation, multiple scattering can be one of several antimicrobial mechanisms to improve photocatalytic performance based on enhanced mass transfer and exchange [18,75]. The bimodal porous self-assembled network of ZnO NPs, with mesopores and macropores was reported to enhance the photocatalytic antimicrobial action caused by multiple scattering under dual UV irradiation [18]. Hierarchically porous self-forming structured aggregates of polydispersed ZnO NPs showed a submicron size of 100–500 nm in diameter, which enhanced the multiple scattering phenomenon [75]. Thus, the hierarchically porous structure of ZnO NPs/MPs mediating multiple scattering can counteract the undesirable photocatalytic performance, which prevents large particles from retaining a small surface area.

Compared to other metal or metal oxide NPs/MPs, ZnO NPs/MPs have multiple functional mechanisms of antimicrobial performance [4]. In antibiotic metals such as silver (Ag) and gold (Au), NPs have the primary functions of metal ion release and membrane adsorption, respectively. In general, titanium dioxide, cupric oxide (CuO), and magnesium oxide (MgO) NPs display ROS generation, NP internalization, and membrane damage, respectively. Regardless of the susceptibility of pathogenic microorganisms to antimicrobial activity, ZnO NPs/MPs can combat microbial regrowth to overcome multi-drug resistance of conventional antibiotics based on targeting antibiotic-resistant pathways in a non-specific manner, irrespective of NP penetration into microorganisms [7,30,76].

Meanwhile, ZnO NP/MP combinations with other materials displayed comparable antimicrobial activity against pathogenic microorganisms [77,78]. In these studies, the materials were mixed or conjugated with ZnO NPs/MPs, with a view to improving their usability and sustainability in biomedical applications, compared to ZnO NPs/MPs alone. Combinatorial materials used with ZnO NPs/MPs also showed antimicrobial activity using different mechanisms to overcome multi-drug resistance. These included antibiotic or anti-inflammatory drugs [20,47,79], other metal oxide NPs/MPs or metal doping [80,81], polymers (e.g., chitosan and alginate) [28,82,83], carbon-based materials (e.g., graphene [84,85], graphene oxide (GO) [86,87], and reduced graphene oxide (rGO) [88]), and quantum dots (QDs) [89,90]. They enhanced ROS generation or mediated the other antimicrobial pathways of ZnO NPs/MPs in microorganisms to create an additive or synergistic microbial growth inhibition benefit. However, the reduced or antagonistic disadvantages of antimicrobial performance sometimes occurred due to microbial susceptibility [20,81,91].

### 3.2. ZnO Materials and Combinations Based on Antimicrobial Functions

ZnO NPs/MPs have been studied to enhance their antimicrobial activity against pathogenic microorganisms [33,92]. In addition to improving their physicochemical characteristics, antimicrobial activities of combinations of ZnO NPs/MPs with other biomaterials have been explored for enhanced antimicrobial performance [93,94]. Table 2 presents the antimicrobial activity of (i) ZnO NPs/MPs, (ii) ZnO NPs/MPs with antibiotic or anti-inflammatory drugs, (iii) ZnO NPs/MPs with other metal oxide NPs/MPs or metal doping, (iv) ZnO NPs/MPs with other polymeric biomaterials or films, and (v) ZnO QDs.

#### 3.2.1. ZnO Materials

The antimicrobial activity of various-sized or -shaped ZnO NPs/MPs has been investigated against pathogenic bacteria as well as viruses [3,69,95]. ZnO NPs/MPs show various forms as spheres, cubes, pyramids, rods, tubes, donuts, platelets, mixtures, and 3D architectures of clusters or networks, ranging from 3.4 nm to 30 μm in size. Size-dependent antimicrobial activity of spherical ZnO NPs of 12–307 nm was described against *S. aureus*, *Proteus vulgaris*, *Salmonella typhimurium*, *Shigella flexneri*, and *Bacillus cereus* [16]. Smaller NPs (12–30 nm) showed a superior antimicrobial growth inhibition (~94.05%) than larger NPs at the same concentration (6 mM). In addition, three types of ZnO NPs shaped like hexagons, spheres, and cubes or rods showed antimicrobial activity against *E. coli*, *S. aureus*, and *B. subtilis* [17]. The minimum inhibitory concentration (MIC) levels of ZnO NPs at ~63 nm, ~65 nm and 60–180 nm, and 40–45 nm were determined as 1562 μg/mL against *E. coil*, 391–781 μg/mL against *S. aureus*, and 195–391 μg/mL against *B. subtilis*, respectively. ZnO nanopyramids at ~15.4 nm in a segment showed an MIC of 333 μg/mL against MRSA [96]. DeLucas-Gil et al. [19] illustrated that nanostructured ZnO of spherical NP clusters at 590 nm in diameter, had superior antimicrobial activity against *E. coli* and *S. aureus* at >3.5 log (control/sample) value than the ZnO NPs at 63 nm in diameter.

ZnO nanorods, at <100 nm or ranging from 500 nm to 1 μm, also showed an antimicrobial activity against pathogenic microorganisms [97,98]. Reddy et al. [99] reported antimicrobial activity of ZnO nanorods at 88.7 nm against *K. pneumoniae*, for which the MIC was 40 μg/mL. These NPs were synthesized by the precipitation method using 2-mercaptoethanol as a capping agent. Tahizdeh et al. [100] reported that ZnO nanorods at an MIC of 250 μg/mL had antimicrobial activity against *E. coli*, *P. aeruginosa*, *S. aureus*, and *Enterococcus faecalis,* excluding *E. faecalis* which was resistant to ZnO nanorods. These nanorods were small crystals 3.4 nm in size and the NPs were 150 nm in length and 21 nm in width (aspect ratio, 7.14). Nanometric ZnO were synthesized using ethylenediamine and citric acid monohydrate as capping agents; the antimicrobial activity of the resulting nanorod-shaped particles (500 nm to 1.0 μm) was affected by the synthesis technique [101]. ZnO nanorods synthesized with ethylenediamine showed antimicrobial activity against *E. coli* but not against *Micrococcus luteus*. Conversely, ZnO nanorods synthesized using citric acid monohydrate showed growth inhibition for *M. luteus* but not for *E. coli*.

Elkady et al. [54] reported antimicrobial activity against *E. coli*, *S. aureus*, *P. aeruginosa*, and *B. subtilis* using as-synthesized ZnO nanopowders of hexagonal hollow tube shapes ~500 nm in length with 17.8 m^2^/g surface area and 278.6 nm average pore size. Their MIC values were found to be 0.0585 mg/mL for *E. coli* (zone of inhibition, 13 mm), 0.234 mg/mL for *S. aureus* (zone of inhibition, 14 mm), 0.234 mg/mL for *P. aeruginosa* (zone of inhibition, 18 mm), and 0.938 mg/mL for *B. subtilis* (zone of inhibition, 15 mm) using disk diffusion. Hollow nanotubes of ZnOs were also reported by López de Dicastillo et al. [55]. They were coated on acrylic polymer/extruded 32 μm-polyethylene (PE) substrate at 1% (wt) and were ~5 μm in length, 59.5 nm in thickness, and 178.2 nm in internal diameter. The hollow nanotubes reduced microbial growth against *E. coli* by 4.67 log (cells/cm^2^) and *S. aureus* by 2.46 log (cells/cm^2^).

Pasquet et al. [58] reported antimicrobial activity by three-different ZnO NPs (ZnO-1, ZnO-2, and ZnO-3) based on differences in the physicochemical characteristics including NP size and morphology of platelets and rods against *E. coli*, *S. aureus*, *P. aeruginosa*, *Candida albicans*, and *Aspergillus brasiliensis*. Platelet-like ZnO NPs (ZnO-1 and ZnO-2) and rod-like ZnO NPs (ZnO-3) formed crystal sizes of 14.7 nm, 17.5 nm, and 76.2 nm, with corresponding surface areas and mesopore sizes of 39.0 m^2^/g and 17.3 nm, 46.9 m^2^/g and 11.6 nm, and 8.25 m^2^/g and 10.6 nm, respectively. The MIC levels of ZnO-1, ZnO-2, and ZnO-3 were 0.12%, 0.18%, and 2.30% (*w/w*) for *E. coli*; 0.25%, 0.30%, and 3.40% (*w/w*) for *S. aureus*; 1.28%, 4.68%, and 5.70% (*w/w*) for *P. aeruginosa*; and >8%, >8%, and >8% (*w/w*) for *C. albicans*, respectively. No growth inhibition was detected against *A. brasiliensis*. Lower MIC levels against microorganisms were detected when using smaller ZnO NPs (ZnO-1 < ZnO-2 < ZnO-3).

Three-dimensional structured ZnO NPs also displayed antimicrobial activity against pathogenic microorganisms [14,18]. Jin et al. [18] presented the antimicrobial activity of self-assembled networks of spherical ZnO NPs at ~3 μm against *E. coli* under dual UV irradiation for 30 s. No colonies were detected at 0.05 mg/mL of self-assembled ZnO networks, whereas colonies at 2.8–3.0 log (CFU/mL) were grown after no particle treatment.

ZnO NP mixtures at 25 nm (1600–3200 μg/mL) particularly had an antimicrobial activity against *P. aeruginosa* isolates from patients which were resistant to amikacin (30 μg), cefepime (30 μg), sparfloxacin (5 μg), piperacillin (100 μg), levofloxacin (5 μg), piperacilin-tazobactum (100/10 μg), imipenem (10 μg), tobramycin (10 μg), nitrofurantoin (300 μg), and ceftazidime (30 μg) [102].

In ZnO MPs, tetrapod-type ZnO NPs which were ~30 μm in size had antimicrobial activity against Herpes simplex virus type-2 (HSV-2) as ZnO tetrapod NPs and HSV-2 cocktail for live virus vaccine [95,103]. ZnO microdonuts were reported by Jeyabharathi et al. [104]. They were nano-to microscale sized donuts at 1–2 μm, and showed growth inhibition against *Enterobacter aerogenes* and *Staphylococcus epidermidis* at 0.5–5 mM.

#### 3.2.2. ZnO Materials with Drugs

Combinations of ZnO NPs/MPs with drugs including antibiotic agents and anti-inflammatory agents have been studied to enhance antimicrobial activity against pathogenic microorganisms [20,21,79,105,106]. Pharmaceutical formulations of ZnO NPs/MPs with antibiotic drugs, anti-inflammatory drugs, or natural products were conventionally used as endodontic dressing (clindamycin and triamcinolone) [107]; dental cements for temporary implants (eugenol) [108,109]; and medical devices or health care products for skin irritation, minor burns, or wounds (aloe vera) [110,111]. In recent approaches to ZnO NP/MP combinations with drugs, various antibiotic drugs were used including azithromycin, gentamicin, oxacillin, cefotaxime, cefuroxime, fosfomycin, chloramphenicol, oxytetracycline [20], cephalexin [21], ciprofloxacin, imipenem [79], ceftriaxone, ceftazidime, gentamicin [105], and ampicillin/sulbactam [106].

The antimicrobial activity of flake ZnO NPs (~200 nm in length) with azithromycin, gentamicin, oxacillin, cefotaxime, cefuroxime, fosfomycin, chloramphenicol, and oxytetracycline, was demonstrated against *E. coli, S. aureus, Salmonella enterica* subsp. *Bukuru*, and *C. albicans* [20]. The combination effects of ZnO NPs with these antibiotics were categorized by synergistic and antagonistic responses compared to ZnO NPs alone at MIC levels of 1.25 mg against *E. coli, S. aureus*, and *S. enterica* subsp. *Bukuru*, excluding *C. albicans,* which had no growth inhibition. Synergistic antimicrobial responses were presented in ZnO NPs (10 mg) with specific antibiotics against microorganisms: azithromycin, oxacillin, cefotaxime, cefuroxime, fosfomycin, and oxytetracycline against *E. coli*; azithromycin, cefotaxime, cefuroxime, fosfomycin, chloramphenicol, and oxytetracycline against *S. aureus*; and oxacillin, cefuroxime, and fosfomycin against *Sallmonella* species. However, ZnO NPs (10 mg) with azithromycin, gentamicin, cefotaxime, neomycin, ampicillin/sulbactam, chloramphenicol, and oxytetracycline had antagonistic antimicrobial responses against *Sallmonella* species. Meanwhile, using ZnO nanohybrids of squeezed ZnO crystals with cephalexin, an MIC level was reported at 1 mg/mL even against *Aeromonas* species [21].

Farzana et al. [79] reported MIC levels of ZnO NPs with ciprofloxacin against *E. coli* and *K. pneumonia* at 0.2–1 mg/mL compared to 0.08 and 0.05 mg/mL for ZnO NPs alone, respectively. However, ZnO NPs with imipenem had no growth inhibition with decreasing antimicrobial effect except for one *K. pneumonia* strain.

Spherical ZnO NPs (15.3–37 nm) with ceftriaxone, ceftazidime, and gentamicin showed growth inhibition against *E. coli, K. pneumonia, S. aureus, E. faecalis, P. aeruginosa*, and *Shigella flexneri* [105]. Compared to ZnO NPs (MIC, 4–16 μg/mL; except *P. aeruginosa*, >64 μg/mL) and antibiotics (MIC, 9–13 μg/mL), after 24 h incubation, ZnO NPs with ceftriaxone, ceftazidime, and gentamicin showed 89–90%, 96–99%, and 95–98% growth inhibition levels against *E. coli, K. pneumonia*, and *S. aureus*, respectively. Growth inhibition levels of 95%, 3.8%, and 96% were also observed against *E. faecalis, P. aeruginosa*, and *S. flexneri*, respectively, for ZnO NPs with ceftriaxone, ceftazidime, and gentamicin.

Sharma et al. [106] described antimicrobial activity of ZnO NPs (25 nm) with ampicillin/sulbactam against *E. coli, K. pneumoniae, P. aeruginosa, S. typhi,* and *S. aureus*. MIC levels of ampicillin/sulbactam were 50 μg/mL against *E. coli, S. typhi*, and *S. aureus*; and 100 μg/mL against *K. pneumoniae* and *P. aeruginosa,* whereas MIC levels of ZnO NPs alone were 25–200 μg. Although the MIC level of ZnO NPs was 25 μg and that of ampicillin/sulbactam was 100 μg/mL against *K. pneumoniae*, the MIC level of the ZnO NP-ampicillin/sulbactam conjugated form against *K. pneumoniae* was 6.25 μg/mL, suggesting antimicrobial activity enhancement.

#### 3.2.3. ZnO Materials with Other Metal Oxide NPs/MPs or Metal Doping

ZnO NPs/MPs have also been studied as combinatorial composites or surface coating agents for enhanced antimicrobial activity against pathogenic microorganisms. Titanium dioxide (TiO_2_) NPs/MPs, 4A zeolite (Na_12_[(AlO_2_)_12_(SiO_2_)_12_]·27H_2_O), and silica (SiO_2_) were included in combinations of ZnO NPs/MPs [112,113,114]. In addition, doping of antibiotic metals (e.g., Ag and Cu) to ZnO NPs/MPs has been reported for enhanced antimicrobial performance [81,105,115,116,117].

Azizi-Lalabadi et al. [80] described growth inhibition of ZnO NPs with TiO_2_ NPs in 4A zeolite against *E. coli* O157:H7, *S. aureus, Pseudomonas fluorescens*, and *Listeria monocytogenes* at MIC levels of 1, 2, 1, and 2 mg/mL, respectively. The ZnO NPs/TiO_2_ NPs/4A zeolites in combinations were cube-shaped at 400–600 nm, in which spherical ZnO NPs with ~50 nm were included. In addition, Ag-ZnO·mSiO_2_ composites reported by Bednář et al. [81], were lamellar porous nanostructures with Ag spots having 250 m^2^/g of specific surface area, and had antimicrobial activity against *E. coli, P. aeruginosa, Streptococcus salivarius, S. aureus*, and *C. albicans* at MIC levels of 2.9 mg/cm^3^, 3.9, 5.9, 5.9, and 23.5 mg/cm^3^, respectively. The Ag-ZnO·mSiO_2_ composites also showed synergistic antimicrobial activity based on Ag and ZnO interaction, compared to ZnO·mSiO_2_ alone. However, ZnO-SiO_2_ composites displayed by Donnadio et al. [115] had antimicrobial activity against *S. aureus* and *C. albicans* at MIC levels of 2 mg/mL (except for ZnO/S-S-20, C-A-10, or C-A-20, >2) as 0.228–0.632 mg/mL of ZnO NPs and 1 or 2 mg/mL (except for ZnO/S-A-10 or S-S-10 >2) as 0.187–0.632 mg/mL of ZnO NPs, respectively. These ZnO-SiO_2_ composites were structured ZnO nanorods of 30–50 nm on the silica surface with specific surface areas of 20–70 m^2^/g.

In 3D porous architectures, CuZnO NPs on mesoporous silica SBA-3 had highly ordered mesoporous structures of near-spherical NPs ~2 μm in size, 3.6274 nm in pore size, and 829 m^2^/g in specific surface area with a relatively rough surface [117]. They showed growth inhibition against *E. coli* and *S. aureus* at MIC levels of 25 mg/mL (0.558 mg/mL as CuZnO NPs) and 6.25 mg/mL (0.139 mg/mL as CuZnO NPs), respectively.

Moreover, ZnO MPs with TiO_2_ MPs showed antimicrobial activity against *E. coli, Streptococcus pyogenes*, and *K. pneumoniae* under UV and visible light irradiation [118]. Using ZnO MPs at 0.25% and TiO_2_ MPs at 1%, growth inhibition levels were 76.8% against *E. coli*, 70.2% against *S. pyrogenes*, and 80.8% against *K. pneumoniae*. ZnO MPs synergistically enhanced antimicrobial activity against pathogenic microorganisms compared to UV and visible light-irradiated TiO_2_ MPs alone. The ZnO MPs in combinations had near-spherical shape, with sizes of 3.17–10.3 μm compared to 2.15–37.1 μm of the TiO_2_ MPs.

#### 3.2.4. ZnO Materials with Other Biomaterials

Antimicrobial polymeric biomaterials or films are used as additive composites in ZnO NP/MP combinations for enhanced antimicrobial activity against pathogenic microorganisms. Recently studied antibiotic biomaterials or films [26,28] in ZnO NPs/MPs combinations included chitosan [28,82,84], silk sericin [84], gelatin [91], gallic acid [28], hydroxyapatite [83,119], alginate [83], polyethyleneglycol (PEG) [120], biopolymer K [27], carrageenan [27], bacterial cellulose [121,122], propolis extract [121], curcumin [84,123,124], graphene [84,85], graphene oxide (GO) [86,87,125], reduced graphene oxide (rGO), and cotton [88,126,127,128,129]. In general, they enhanced the antimicrobial activity of ZnO NPs/MPs and overcame formulation challenges of ZnO NPs/MPs in manufacturing beads or films and covering surfaces for coating.

Among natural products, chitosan has been extensively studied as a pharmaceutical excipient for drug delivery carriers in biomedical applications owing to their biocompatibility and physicochemical characteristics [113,114]. It is beneficial to have inherent therapeutic functions including antimicrobial activity and anti-inflammatory activity for accelerated wound healing, hemostasis, and reduced fat absorption to decrease plasma and liver lipids and increase fecal excretion [114,130]. Regarding the biomedical functions of chitosan, the antimicrobial mechanisms are explained by cell binding via charge–charge interaction owing to the polycationic nature of chitosan, leading to disruption of the membrane and inhibition of nucleic acid processes, thereby causing cell death. As a chelating agent, chitosan also interacts with trace metal elements, which are toxic to cells, inducing microbial growth inhibition. It is applied for wound dressing/healing, cotton fabric, daily food packaging, and paper packaging as an antimicrobial agent, mostly in films [113,131,132,133,134]. Therefore, chitosan can be extended to the combinations with ZnO NPs/MPs [135].

Chitosan-ZnO NP-loaded gallic acid (C-ZnO@gal) films showed antimicrobial activity against *E. coli* and *B. subtilis* [28]. ZnO@gal NPs were near-rod-shape at ~19.2 nm, and C-ZnO@gal films included homogeneously distributed ZnO@gal NPs on chitosan matrixes (2 g chitosan/70 mg ZnO@gal) at a thickness of 0.10 mm. Using C-ZnO@gal films at 0.5 mg/mL, growth inhibition zone levels were reported as 28 mm against *E. coli* and 25 mm against *B. subtilis*. Al-Nabulsi et al. [82] described the antimicrobial activity of ZnO NP-containing chitosan against *E. coli* O157:H7, which was used as a biocompatible coating agent for smart storage. The ZnO NPs had a near-spherical shape at ~65 nm and were uniformly distributed in chitosan matrixes. The growth reduction levels against *E. coli* O157:H7 at 4 °C were 2.5 log (CFU/g) for chitosan (2.5%, *w/v*) and 2.8 log (CFU/g) for ZnO NP (1%, *w/v*)-containing chitosan (2.5%, *w/v*). Although combinations of ZnO NPs in a chitosan matrix did not show a significant improvement of antimicrobial activity against *E. coli* O157:H7 (*p* > 0.05) compared to ZnO NPs or chitosan alone, the ZnO NP combination in the chitosan matrix presented better antimicrobial results.

Kumar et al. [91] reported growth inhibition levels of chitosan/gelatin hybrid nanocomposite films containing ZnO NPs against *E. coli* and *S. aureus* along with their improved structural integrity. In these films, ZnO NPs had various shapes such as polyhedrons, quasi-spheres, or rods of 20–40 nm, 500–1000 nm, and 200–400 nm, respectively, which were evenly distributed on smooth, compact, and heterogeneous surfaces with 86–92 μm in thickness. Depending on the ZnO NP contents in the films, their antimicrobial activity against *E. coli* presented a 10.5 mm zone of growth inhibition for ZnO NPs at 1% and 2% in the films and a 10.7 mm zone of growth inhibition for ZnO NPs at 4% in the films. The films showed comparable growth inhibition results based on inherent antimicrobial activity of chitosan. Unlike the effects against *E. coli,* no prominent results were obtained for *S. aureus*.

Three-dimensional porous ZnO NP scaffolds with chitosan/silk/sericin for wound dressing/healing were described as antimicrobial agents against *E. coli* and *S. aureus* [136]. Their porous microstructures (1.5 × 1.5 cm^2^) comprising 2% (*w/v*) chitosan, and 100 μL or 250 μL of ZnO NPs (40% dispersion, wt), showed ~86% porosity with 4–200 μm in pore size. Based on the zone of growth inhibition via the disk diffusion method for evaluation, these particles displayed 2–4.5 mm growth inhibition against *E. coli* and 2.5–5.5 mm growth inhibition against *S. aureus*.

In particular, Javed et al. [137] reported a biodental materials for orthodontics, composed of star-shaped, chitosan-based ZnO NPs (20–25 nm), which showed enhanced antimicrobial activity against *K. pneumoniae* (13 mm zone of inhibition, the highest); *E. coli*, *P. aeruginosa*; and *B. subtillis*, *S. aureus*, and MRSA (6 mm zone of inhibition, the lowest), compared to those composed of ZnO NPs or chitosan alone. In the synthesis of chitosan-based ZnO NPs via co-precipitation, zinc acetate dihydrate (0.06 M) and chitosan were used as a precursor and a capping agent, respectively. Conventionally used adhesive was coated using chitosan-based ZnO NPs at 2–10%. The pure ZnO NPs had a spherical shape with a diameter of 25–30 nm.

Meanwhile, hydroxyapatite-biphasic ZnO NP/MP-embedded alginate (HA-ZnO-Alg) beads showed antimicrobial activity against *E. coli, P. aeruginosa, S. aureus*, and *S. epidermidis* [83]. In alginate beads, ZnO NPs/MPs had a snowflake shape at <1 μm, which were evenly distributed with hydroxyapatite in alginate bead matrixes. Antimicrobial growth inhibition levels of HA-ZnO-Alg beads at 0.1 mg/mL were 56% against *E. coli*, 65% against *P. aeruginosa*, and 100% against *S. aureus* and *S. epidermidis*.

Jose et al. [120] also reported growth inhibition of tungsten-doped polyethylene glycol (PEG)-capped ZnO (W-PEG-ZnO) NPs against *E. coli* and *B. cereus*, which were near-spherical at ~40.46 nm. Compared to ampicillin at 100 μg/μL inhibiting growth at 35 mm against *E. coli* and 20 mm against *B. cereus*, W-PEG-ZnO NPs showed a 5–6 mm zone of growth inhibition against *E. coli* at 400–600 μg/μL and a 6–8 mm zone of growth inhibition against *B. cereus* at 300–600 μg/μL, suggesting *B. cereus* was more susceptible to W-PEG-ZnO NPs than *E. coli*.

Antimicrobial activity of biopolymer K-carrageenan-wrapped ZnO (KC-ZnO) NPs, oval-shaped at 97 nm, was reported against MRSA [27]; the MIC level of KC-ZnO NPs was 7.5 μg/mL. ZnO NP films manufactured with bacterial cellulose and propolis extract resulted in quasi-spherical ZnO NPs (70–90 nm), homogeneously distributed on the substrate surface in bacterial cellulose-ZnO NP-propolis extract (BC-ZnO-propolis) films [121]. The BC-ZnO-propolis films had antimicrobial activity against *E. coli* at an MIC level of >1.89 mg/mL, *B. subtilis* at 0.44 or <0.44 mg/mL, and *C. albicans* at >0.8, 1.3, 1.89, and >1.89 mg/mL, depending on ZnO NP types according to ultrasound frequency (40 kHz and 100 kHz) during the synthesis, and propolis extract contents (3.5%, 7%, 11%, and 15%, wt).

Curcumin in turmeric is known to have beneficial anti-inflammatory activity, and is used as a multipurpose nutraceutical in various dosage forms of tablets, capsules, and ointments [123]. After loading curcumin on ZnO (C-ZnO) NPs, the antimicrobial activity of C-ZnO NPs against *E. coli*, *S. epidermis*, *S. aureus*, and *B. cereus* was determined [124]. Among the C-ZnO NPs, spheres (C-sZnO), rods (C-rZnO), javelin (C-jZnO), short petal nanoflowers (C-spZnO), and long petal nanoflowers (C-lpZnO) were detected with sizes of 40–100 nm, 600–900 nm (<300 nm of width), 300–600 nm (<300 nm of width), 2–4 μm, and ~500 nm, respectively. Their microbial growth inhibition levels were confirmed via measurements of well diffusion zones of inhibition as follows: C-sZnO, 8.4 mm; C-rZnO, 10.1 mm; C-jZnO, 11.1 mm (the highest); C-spZnO, 8.1 mm; C-lpZnO, 9.8 mm; and curcumin, 7.4 mm (the lowest) against *E. coli*; C-sZnO, 19.1 mm; C-rZnO, 17.2 mm; C-jZnO, 19.4 mm; C-spZnO, 16.4 mm; C-lpZnO, 20.1 mm (the highest); and curcumin, 8.2 mm (the lowest) against *S. epidermis*; C-sZnO, 15.4 mm; C-rZnO, 16.6 mm; C-jZnO, 13.4 mm; C-spZnO, 15.2 mm; C-lpZnO, 17.0 mm (the highest); curcumin, 8.1 mm (the lowest) against *S. aureus*; and C-sZnO, 17.4 mm; C-rZnO, C-jZnO, 18.7 mm (the highest); C-spZnO, 14.2 mm; C-lpZnO, 14.2 mm; and curcumin, 8.4 mm (the lowest) against *B. cereus*.

Graphene, graphene oxide (GO), and reduced GO (rGO) have also been studied as antibiotic agents in combinations of ZnO NPs with other biomaterials. Antimicrobial activity of graphene/ZnO nanocomposite films against *Streptococcus mutans* was reported by Kulshrestha et al. [84]. The ZnO NPs distributed in the graphene sheet films were near-spherical at 20–40 nm. The MIC level of graphene/ZnO nanocomposite films was 125 μg/mL. Oves et al. [85] described the antimicrobial activity of graphene/ZnO nanocomposites with curcumin against MRSA strains (ATCC 43300 and ATCC BAA-1708). In the nanocomposites, spherical ZnO NPs at 35 nm were homogenously distributed on graphene sheets. Compared to curcumin with an MIC of 125 μg/mL, the graphene/ZnO nanocomposite induced growth inhibition at 125 μg/mL against MRSA ATCC 43300 and at 250 μg/mL against MRSA ATCC BAA-1708. Use of curcumin together with the graphene/ZnO nanocomposites achieved growth inhibition against MRSA ATCC 43300 and MRSA ATCC BAA-1708 at 31.25 and 62.5 μg/mL, respectively. Against in vivo topical dermatitis infection, the growth of MRSA was inhibited by up to 64%.

GO/ZnO nanocomposites for wound care showed growth inhibition against *E. coli, S. typhi, P. aeruginosa,* and *S. flexneri* [86]. GO with smooth and wrinkled surface layers was used to prepare GO/ZnO nanocomposites. The ZnO NPs were well incorporated and distributed on the GO sheets generating agglomerates. Analysis of the zone of growth inhibition with GO/ZnO nanocomposites at levels of 25–100 μg/mL under dark or visible light irradiation, showed that the composites were more susceptible to *E. coli, P. aeruginosa, S. typhi*, and *S. flexneri* than with GO alone. In addition, Wang et al. [87] reported antimicrobial activity against *E. coli* of GO/ZnO composites, including rod-like ZnO NPs at 4 nm, homogenously attached on GO sheets. Using 1–4 μg GO/ZnO composites, growth inhibition zones were detected at 2 and 4 μg (MIC, 2 μg).

In the case of rGO, combination of ZnO NPs with rGO had antimicrobial activity against microorganisms [88]. Spherical ZnO NPs at 100–300 nm were attached to rGO sheets for manufacturing rGO/ZnO films with roughness at 159 nm, containing homogenously distributed ZnO NPs on rGO sheets. They displayed >99% (2-log) growth reduction against *S. aureus* at 1% (wt) rGO.

In cotton fabrics, ZnO NPs have been used as protective agents against textile degradation, unpleasant odor, and potential health risks caused by microbial growth after contact with the human body [128,130,138]. As an alternative to chemical biocides, ZnO NPs provide enhanced antimicrobial activity against pathogenic microorganisms in surface-modified textiles during the manufacturing process. Noman, M.T. and M. Petrů [127] reported eight types of cotton-ZnO NP (C-nZnO) composites and their antimicrobial actions against *E. coli* and *S. aureus*. The ZnO NPs were spherical at 27 nm, and formed thick and dense layers on cotton surfaces in C-nZnO composites at 2.2%, 1.7%, 4.9%, 4.3%, 11.1%, 7.8%, 22.2%, and 16.7% (wt). They showed microbial growth reduction of 97–100% against *E. coli* and 96–98% against *S. aureus*. Subash et al. [129] also demonstrated microbial growth reduction by ZnO NP-coated fabric against *E. coli* and *S. aureus*. The ZnO-coated fabric comprised evenly distributed spherical ZnO NPs (200 nm) on the fabric surface. In a 4.8 cm-diameter circular cut of the fabric, *E. coli* and *S. aureus* growth levels were reduced by 80% and 99.99%, respectively. In the case of ZnO MPs, they were coated on cotton fabrics together with chitosan and formed a uniformly distributed dense microstructure of rods showing antimicrobial growth inhibition against *E. coli* [126]. Immersion of ZnCl_2_ (4%) for ZnO MP synthesis on chitosan-loaded cotton fabrics (1–2%/1 g cotton fabric) resulted in a growth inhibition zone of 2.5 cm in disk diffusion against *E. coli*.

#### 3.2.5. ZnO QDs

ZnO QDs have been studied for imaging and therapy as theranostic agents having photoluminescence properties with antimicrobial activity against pathogenic microorganisms [139,140]. They have superior characteristics including high quantum yield, high stability, and a narrow emission range.

Based on different nanostructures of nanorods/nanotubes (ZnO QD-1, ZnO QD-2, and ZnO QD-12), nanospheres (ZnO QD-3, ZnO QD-4, ZnO QD-6, ZnO QD-7, ZnO QD-8, ZnO QD-10, ZnO QD-11, and ZnO QD-14), nanowhiskers (ZnO QD-5), nanoflowers, and mixtures of nanorods and nanoflowers (ZnO QD-13) (ZnO QD-9: not mentioned), the growth inhibition levels of 14 ZnO QDs were evaluated against *E. coli, K. pneumonia, P. aeruginosa, S. aureus, L. monocytogenes, E. faecalis, E. aerogenes, B. anthracis, B. cereus*, and *S. epidermidis* [89]. The lowest MIC levels among the ZnO QDs from ZnO-1 to ZnO-10 were as follows: ZnO QD-1, 25 mg/mL against *E. coli*; ZnO QD-4 and ZnO QD-6, 25 mg/mL against *E. aerogenes*; ZnO QD-3, and ZnO QD-5, 12.5 mg/mL against *K. pneumonia*; ZnO QD-3 and ZnO QD-7, 12.5 mg/mL against *P. aeruginosa*; ZnO QD-2, ZnO QD-3, and ZnO QD-8, 6.25 mg/mL against *B. anthracis*; ZnO QD-8, 6.25 mg/mL against *S. aureus*; ZnO QD-6 and ZnO QD-7, 50 mg/mL against *L. monocytogenes*; ZnO QD-2 and ZnO QD-7, 25 mg/mL against *E. faecalis*; ZnO QD-3 and ZnO QD-5, 12.5 mg/mL against *B. cereus*; and ZnO QD-8, 1.5 mg/mL against *S. epidermidis*. Singh et al. [90] reported spherical ZnO QDs of ~6 nm that showed an inhibition zone of 15.69 mm against *E. coli*. Compared to the growth inhibition level at 5 mM of bulk zinc acetate, that of ZnO QDs against *E. coli* increased by 1.6-fold.

ZnO QDs are also functionalized with polymers, peptides, and antibiotic drugs [140,141,142]. Antimicrobial peptide (BSA-PEP-MPA) was attached to ZnO QDs (spheres, 104 nm) containing vancomycin (Van@ZnO-BSA-PEP-MPA) and methicillin (Met@ZnO-BSA-PEP-MPA) for enhanced antimicrobial activity against *S. aureus, B. subtilis*, and MRSA [141]. Analysis of MIC levels showed that ZnO@BSA-PEP-MPA had no growth inhibition potential, used as a nanoprobe promoting permeability of antibiotics through microbial membrane. In the case of Van@ZnO-BSA-PEP-MPA, 2.0 μg/mL against *S. aureus* and 1.0 μg/mL against *B. subtilis* were detected as MIC levels. Growth inhibition at 6 × 10^4^ log (CFU/g) was induced after administration of 4 × 10^8^ CFU of *S. aureus* for infection and 5.0 mg/kg of Van@ZnO-BSA-PEP-MPA as *in vivo* diagnostic agent with no changes in activity and body weight for theranostics. However, Met@ZnO-BSA-PEP-MPA showed 64 μg/mL of MIC against MRSA. Polyvinylprolidone-capped ZnO (PVP-ZnO) QDs also showed antimicrobial activity against *E. coli* O157:H7, *S. enteritidis*, and *L. monocytogenes*. Spherical ZnO QDs of ~5 nm were used to prepare PVP-ZnO QDs, which were highly crystalline spheres at 4 nm (<ZnO QDs) [142]. Compared to ZnO QDs at 1.12 mg/mL mediating growth inhibition of 63.9% against *S. enteritidis* and 80.6% against *L. monocytogenes*, PVP-ZnO QDs at 40 mg/mL showed growth inhibition of 66.7% against *E. coli* O157:H7 and 58.9% against *L. monocytogenes*.

## 4. Current Biomedical Applications

**Figure 2:**
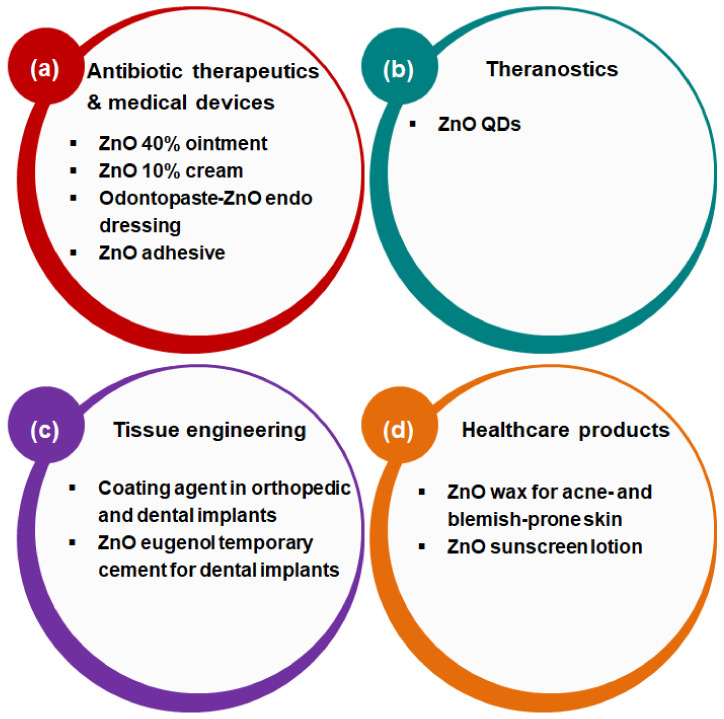
Functional approaches of ZnO materials for biomedical applications: (**a**) antibiotic therapeutics and medical devices, (**b**) theranostics, (**c**) tissue engineering, and (**d**) healthcare products.

ZnO NPs/MPs are one of Zn-based materials that have been extensively used in various biomedical fields including antibiotic therapy, medical devices, theranostics, tissue engineering, and health care because of their antimicrobial functions against pathogenic microorganisms (Figure 2) [11,143,144,145,146,147,148]. In the human body, Zn plays a pivotal role in life cycle maintenance and Zn deficiency causes cell impairment and malignancy leading to severe diseases related to immune responses such as infection and cancer [69]. As a first aid antibiotic alternative for the protection of skin [143,147], ZnO ointment (40%) or cream (10%) is conventionally used. In addition, ZnO NPs/MPs and antibiotic drugs are applied as endo dressing in paste forms [107]. They are also used as ZnO surgical adhesives in medical devices [144,145]. Moreover, ZnO NPs/MPs have been utilized as postoperative dressing on leg excisions [148]. In theranostics, biocompatible ZnO QDs are explored as photodynamic therapeutics as well as diagnostic agents owing to their photoluminescence properties based on photochemical stability [11]. For dental tissue engineering, ZnO NPs/MPs are used as coating agents in orthopedic and dental implants [146]. ZnO NPs/MPs and eugenol are also extensively used as temporary cement for dental implants [108]. ZnO waxes or sunscreen lotions are used as health care products for skin protection against acne/blemish or UV and visible light [149,150]. Although ZnO NPs/MPs still have exposure risks to health via the inhalation route [151,152], ZnO is considered as “generally recognized as safe” and is approved by the U.S. Food and Drug Administration and the health risks of ZnO NPs/MPs are guided by the Organisation for Economic Co-operation and Development, based on their unique physicochemical characteristics via synthesis techniques [153]. Therefore, ZnO NPs/MPs have a promising potential for use in biomedical applications including medicine, medical devices, and cosmeceuticals connected with antibiotic functions overcoming drug resistance.

## 5. Conclusions

The antibiotic properties of ZnO materials have been highlighted for their biomedical applications in combating multi-drug resistance. Compared to currently available antibiotic drugs, they exhibit different mechanisms of antimicrobial actions; these mechanisms are mainly induced by the Zn^2+^ ion, particle adsorption, ROS generation and photocatalytic responses based on physicochemical characteristics of these materials, which vary substantially according to their synthesis techniques. ZnO NPs, sometimes MPs, show enhanced antimicrobial performance even toward pathogenic viruses. Their combinations with other antibiotic drugs, metal oxide NPs/MPs or metal doping, and other biomaterials also have a wide-spectrum antimicrobial activity that can be customized for multi-therapeutic options and used to improve their applicability as industrial and clinical translation platforms. Moreover, ZnO QDs display enhanced antimicrobial actions as theranostic agents. Therefore, ZnO materials can be a next-generation antibiotic drug against multi-drug resistant pathogenic microorganisms and, in the near future, combinations of these can be developed further with industrial and clinical impacts.

## Figures and Tables

**Table 1:** Physicochemical characteristics of zinc oxide (ZnO) materials.

Morphology	Particle Size	Porosity	Surface Area (m^2^/g)	Synthesis Method (Precursor)	Refs.
Spheres	12 nm, 25 nm	Slit-like pores	90.4, 42.8	▪ Solvothermal synthesis using tetramethyl ammonium hydroxide (TMAOH) (zinc nitrate hexahydrate)	[16]
	62–94 nm	Not available	Not available	▪ Green synthesis using Catharanthus roseus leaf extract (0.01 M zinc acetate dehydrate)	[47]
	15–20 nm	Not available	Not available	▪ Green synthesis & co-precipitation using Bambusa Vulgaris leaf extract (0.5 mol/50 mL zinc nitrate in water)	[48]
	25–30 nm (TEM); 65 ± 4 nm (dynamic light scattering)	Not available	Not available	▪ Green synthesis using Sambucus ebulus leaf extract (1 M zinc acetate dihydrate)	[49]
	12–46 nm	Not available	Not available	▪ Green synthesis using Hibiscus subdariffa leaf extract with further heating at 60 °C for 4 h (91 mM zinc acetate in water)	[50]
Spheres in self-assembled network	48.3 nm	Mesopores, 5–6.25 nm; macropores, 2–6 μm	Not available	▪ Sol–gel synthesis using oleylamine with annealing process at 150 °C (zinc acetylacetonate dihydrate)	[18]
Spheres in NP cluster	NPs-63 nm; cluster-590 nm	34 nm	20.72	▪ Soft chemical synthesis using urea (3.6 mol) and glycerol (3.6 mol) (micrometric ZnO, 6 wt.%)	[19]
Dumbbells	Length 190–250 nm; breadth 50–60 nm	Not available	Not available	▪ Green synthesis using Hibiscus subdariffa leaf extract, dried at 100 °C for 4 h (91 mM zinc acetate in water)	[50]
Nanorods	Length 523 nm; diameter 47 nm	Not available	2.3	▪ Sol–gel synthesis using 1 M hexamethyl tetraamine (HMTA) at pH 6.5 (1 M zinc nitrate in water)	[52]
	Length ~2 μm; diameter ~50 nm	Not available	Not available	▪ Green synthesis using Egg white albumen calcined at 650 °C (1 mmol/mL zinc acetate dihydrate)	[53]
Hollow nanotubes	Length ~500 nm	53 nm (900 °C annealing), 114.6 nm (700 °C annealing), 255.4 nm (500 °C annealing), 278.6 nm (as-synthesized)	2.4 (900 °C annealing), 7.2 (700 °C annealing), 16.7 (500 °C annealing), 17.8 (as-synthesized)	▪ Hydrothermal synthesis using 0.25 mM polyvinyl alcohol (PVA) at pH 9 with annealing process at 500–900 °C (14 mM zinc acetate dihydrate)	[54]
	Length ~5 μm; thickness 59.5 nm	Internal diameter 178.2 nm	Not available	▪ Atomic layer deposition (ALD) over electrospun polyvinyl alcohol (PVA) nanofibers (template) after polymer removal through calcination or hydrolysis (diethyl zinc (Zn(C2H5)2)	[55]
Needles	Length ~2 μm; diameter 20–40 nm	Not available	Not available	▪ Green synthesis using Berberis aristata leaf extract, adjusting pH with 1 M sodium hydroxide (NaOH) (0.1 M zinc acetate dihydrate)	[56]
Disks	41 nm	Not available	Not available	▪ Co-precipitation using 2 M sodium hydroxide in water at room temperature (1 mM zinc acetate dihydrate)	[57]
Platelets	14.7 nm, 17.5 nm; hydrodynamic environment- 6.4 μm, 5.0 μm	17.3 nm, 11.6 nm	39.0, 46.9	▪ Not available (conventional products)	[58]
Flowers	Length 200–500 nm; length 1–2 μm	Not available	Not available	▪ Electrochemical-thermal technique using poly-diallyl-(dimethylammonium) chloride (PDDA) and benzyl-dimethylammonium chloride (BAC) as a stabilizer (Two Zn electrodes)	[45]
	23.7–88.8 nm	Not available	Not available	▪ Sol–gel synthesis using 0.2 *w/v*% cetyltrimethyl ammonium bromide (CTAB) at pH 11.7–11.9, and near-room temperatures (25–75 °C) (5% zinc acetate dihydrate)	[59]
	700 nm–2.2 μm	Not available	Not available	▪ Co-precipitation using 0.1 M NaOH calcined at 550 °C (0.1 M zinc nitrate hexahydrate)	[60]
	45 nm	Not available	28.8	▪ Solvothermal synthesis using 0.5 M NaOH at pH 9 (5.60 g/50 mL zinc acetate dihydrate in water)	[61]
Stars	31 nm	Not available	Not available	▪ Liquid precipitation using 1 M NaOH (0.01 mol/50 mL zinc acetate dihydrate in water)	[62]
Flakes	32 nm	Not available	Not available	▪ Co-precipitation using 0.02 M sodium carbonate (0.01 M zinc acetate)	[63]

**Table 2:** Antimicrobial activity of zinc oxide (ZnO) materials against pathogenic microorganisms.

Materials	Synthesis Techniques (Precursor)	Material Description (Morphology/Particle Size)	Antimicrobial Activity Test Methods	Antimicrobial Activity against Pathogenic Microorganisms	Refs.
**ZnO materials**					
0D structured ZnO NPs	Hydrothermal method or room temperature synthesis (zinc nitrate hexahydrate; zinc sulfate; zinc acetate dihydrate)	Spheres/12 nm, 25 nm; 30 nm, 88 nm, 142 nm, 212 nm, 307 nm	Turbidity	6 mM, [*Staphylococcus aureus*] 30 nm: 10.11% growth; 88 nm: 54.34% growth; 142 nm: 78.12% growth; 212 nm: 79.79% growth; 307 nm: 96.67% growth, [*Proteus vulgaris*] 12 nm: 8.71% growth, [*Salmonella typhimurium*] 12 nm: 46.96% growth, [*Shigella flexneri*] 12 nm: 5.95% growth, [*Bacillus cereus*] 12 nm: 7.62% growth	[16]
	Green method using *Aloe vera* leaf extract (zinc acetate dihydrate)	▪ ZnO-1 & ZnO-2: hexagons and spheres/~63 nm; ~65 nm, and 60–180 nm ▪ ZnO-3: cubes or rods/40–45 nm	Well diffusion, broth microdilution; MIC	MIC, [*E. coli*] ZnO-1, ZnO-2, and ZnO-3: 1562 μg/mL; [*S. aureus*] ZnO-1: 781 μg/mL, ZnO-2 and ZnO-3: 391 μg/mL; [*B. subtilis*] ZnO-1 and ZnO-2: 391 μg/mL; ZnO-3: 195 μg/mL	[17]
	Precipitation method (zinc acetate dihydrate)	Nanopyramids/~15.4 nm in segments	Colony counting, serial dilution; MIC	[Methicillin-resistant *S. aureus* (MRSA)] MIC, 333 μg/mL (3 log (CFU/mL) inhibition at 800 μg/mL)	[96]
	Soft chemical synthesis (micrometric ZnO)	Spherical NP cluster/cluster-590 nm; NPs-63 nm	Colony counting	Antimicrobial activity value of log (control/sample), [*E. coli*] > 3.5; [*S. aureus*] > 3.5	[19]
1D structured ZnO NPs	Hydrothermal method (zinc acetate dihydrate)	Hollow nanotubes/particle size ~500 nm in length; surface area 17.8 m^2^/g; average pore size, 278.6 nm	Disk diffusion; MIC	As-synthesized ZnO nanopowders, MIC, [*Escherichia coli*] 0.0585 mg/mL, [*S. aureus*] 0.234 mg/mL, [*Pseudomonas aeruginosa*] 0.234 mg/mL, [*Bacillus subtilis*] 0.938 mg/mL	[54]
	Atomic layer deposition process over polymer template and template removal	Hollow nanotubes/length, ~5 μm; thickness, 59.5 nm; internal diameter, 178.2 nm	Colony counting	ZnO nanotubes at 1 wt% with acrylic polymer/extruded 32 μm-polyethylene (PE) substrate film coating [*E. coli*] 4.67 log reduction (cells/cm^2^), [*S. aureus*] 2.46 log reduction (cells/cm^2^)	[55]
	Precipitation method (zinc nitrate)	Nanorods/88.7 nm using 2-mercaptoethanol as a capping agent	Disk diffusion method, serial dilution; MIC	[*Klebsiella pneumoniae*] MIC, 40 μg/mL	[99]
	Green method using *Chlorella vulgaris* culture supernatant (zinc acetate dihydrate)	Nanorods/crystal size, 3.4 nm; length-150 nm; width-21 nm (aspect ratio of length to width 7.14)	Microdilution; MIC	[*E. coli, P. aeruginosa, S. aureus*] MIC, 250 μg/mL (excluding *Enterococcus faecalis*: resistant to ZnO nanorods)	[100]
	Sol–gel method (zinc nitrate hexahydrate)	Nanorods/500 nm–1 μm	Disk diffusion	[Capping agent: ethylenediamine] *E. coli*-inhibition, *Micrococcus luteus*-no inhibition, [Capping agent: citric acid monohydrate] *E. coli*-no inhibition, *B. subtilis*-inhibition	[101]
2D structured ZnO NPs	Not available (conventional products)	▪ ZnO-1: platelets/14.7 nm ▪ ZnO-2: platelets/17.5 nm ▪ ZnO-3: rods, 76.2 nm	Disk diffusion, broth dilution; MIC	ZnO-1, ZnO-2, ZnO -3, MIC (wt%), [*E. coli*] 0.12, 0.18, 2.30; [*S. aureus*] 0.25, 0.30, 3.40; [*P. aeruginosa*] 1.28, 4.68, 5.70; [*Candida albicans*] >8; >8; >8; [*Aspergillus brasiliensis*] nil, nil, nil	[58]
3D structured ZnO NP network	Sol–gel method (zinc acetylacetonate hydrate)	Spherical NP aggregate network/aggregates: ~3 μm; NPs: 48.3 nm	Colony counting; photocatalytic incubation	[*E. coli*] {Dual UV for 30 s} no particles-~approx. 2.8–3.0 log (CFU/mL), 0.05 mg/mL-no colonies detected	[18]
ZnO NP mixtures	Green method using *Butea monsoperma* seed extract (zinc nitrate hexahydrate)	Mixtures/25 nm	Broth dilution; MIC	[*P. aeruginosa* (resistant to amikacin (30 μg), cefepime (30 μg), sparfloxacin (5 μg), piperacillin (100 μg), levofloxacin (5 μg), piperacilin-tazobactum (100/10 μg), imipenem (10 μg), tobramycin (10 μg), nitrofurantoin (300 μg), and ceftazidime (30 μg))] MIC, 1600 μg/mL; 1600–3200 μg/mL for isolates from patients	[102]
ZnO MPs	Flame transport synthesis (zinc microparticles)	Tetrapods/~30 μm	Plaque assay	[Herpes simplex virus type-2 (HSV-2)] ZnO tetrapod NP and HSV-2 cocktail for live virus vaccine	[95,103]
	Green method using starch (zinc acetate)	Self-assembled hollow microdonuts/1–2 μm	Disk diffusion	[*Enterobacter aerogenes* and *Staphylococcus epidermidis*] 0.5–5 mM	[104]
**Combinations of ZnO materials with drugs**					
ZnO NPs with azithromycin, gentamicin, oxacillin, cefotaxime, cefuroxime, fosfomycin, chloramphenicol, and oxytetracycline	Green method using *Ulva fasciata* alga extract (zinc acetate dehydrate)	Flakes/length, ~200 nm	Disk diffusion; MIC	▪ ZnO NPs: MIC, [*E. coli*, *S. aureus*, and *Salmonella enterica* subsp. *Bukuru*] 1.25 mg [*C. albicans*] no inhibition ▪ ZnO NPs (10 mg) with antibiotics: [*E. coli*] azithromycin, oxacillin, cefotaxime, cefuroxime, fosfomycin, oxytetracycline—synergistic, [*S. aureus*] azithromycin, cefotaxime, cefuroxime, fosfomycin, chloramphenicol, oxytetracycline—synergistic, [*Salmonella* spp.] oxacillin, cefuroxime, fosfomycin—synergistic, azithromycin, gentamicin, cefotaxime, neomycin, ampicillin/sulbactam, chloramphenicol, oxytetracycline—antagonistic	[20]
ZnO with cephalexin nanohybrids	Ion exchange via sol–gel method (ZnO, and 2,4-chlorophenoxyacetic acid)	Squeezed ZnO crystals/not available	Disk diffusion; MIC	ZnO with cephalexin nanohybrids: MIC, [*Aeromonas* spp.] 1 mg/mL	[21]
ZnO NPs with β-lactam antibiotics (ciprofloxacin and imipenem)	Not available (conventional product)	Not available	Disk diffusion; agar dilution; MIC	MIC ▪ ZnO NPs: [*E. coli*] 0.08 mg/mL, [*K. pneumonia*] 0.05 mg/mL ▪ ZnO NPs with ciprofloxacin: 0.2–1 mg/mL ▪ ZnO NPs with imipenem: decrease in antimicrobial effect (except for one *K. pneumonia* strain)	[79]
ZnO NPs with ceftriaxone (CFX-ZnO NPs), ceftazidime (CFZ-ZnO NPs), and gentamicin (GTM-ZnO NPs)	Green method using *Enterococcus faecalis* culture supernatant (zinc sulfate)	Spheres/15.3–37 nm	Broth dilution; MIC	▪ ZnO NPs: [*E. coli, K. pneumonia, S. aureus, E. faecalis, P. aeruginosa*, and *S. flexneri*] MIC, 4–16 μg/mL (*P. aeruginosa* > 64) ▪ Antibiotics: [*E. coli, K. pneumonia*, *S. aureus, E. faecalis, P. aeruginosa*, and *S. flexneri*] MIC, 9–13 μg/mL ▪ 24 h incubation: [*E. coli*] CFX-ZnO NPs 89–90% inhibition, [*K. pneumonia*] CFZ-ZnO NPs 96–99% inhibition, [*S. aureus*] GTM-ZnO NPs 95~98% inhibition, [*E. faecalis*] GTM-ZnO NPs 95% inhibition, [*P. aeruginosa*] CFZ-ZnO NPs 3.8% inhibition, [*S. flexneri*] CFZ-ZnO NPs 96% inhibition	[105]
ZnO NPs with ampicillin/sulbactam	Milling method (zinc chloride)	Not available/25 nm	Disk diffusion; broth dilution; MIC	MIC, ▪ Ampicillin/sulbactam: [*E. coli, S. typhi*, and *S. aureus*] 50 μg/mL, [*K. pneumoniae*, and *P. aeruginosa*] 100 μg/mL ▪ ZnO NPs: 25–200 μg ▪ ZnO NPs with ampicillin/sulbactam: [*K. pneumoniae*] 25 μg + 100 μg/mL, respectively ▪ ZnO NP-ampicillin/sulbactam (conjugated form): [*K. pneumoniae*] 6.25 μg/mL	[106]
**Combinations of ZnO materials with other metal oxide NPs** **/** **MPs, or metal doping**					
ZnO NPs with TiO_2_ NPs in 4A zeolite	Ion exchange process (zinc acetate dehydrate)	Cubes/400–600 nm (NPs: spheres, ~50 nm)	Disk diffusion; MIC	MIC, [*E. coli* O157:H7] 1 mg/mL, [*S. aureus*] 2 mg/mL, [*Pseudomonas fluorescens*] 1 mg/mL, [*Listeria* *Monocytogenes*] 2 mg/mL	[80]
Ag-ZnO·mSiO_2_ composites	Precipitation of sodium water glass in zinc acetate solution (zinc acetate dihydrate)	Lamellar porous nanostructure with silver spots/not available (specific surface area, 250 m^2^/g)	Microdilution; MIC	MIC, [*E. coli*] 2.9 mg/cm^3^, [*P. aeruginosa*] 3.9 mg/cm^3^, [*Streptococcus salivarius*] 5.9 mg/cm^3^, [*S. aureus*] 5.9 mg/cm^3^, [*C. albicans*] 23.5 mg/cm^3^—synergistic	[81]
ZnO-SiO_2_ composites	Solid state mixing (zinc acetate dihydrate)	▪ Nanostructures on the silica surface/not available ▪ NPs: nanorods/30–50 nm (specific surface area, 20–70 m^2^/g)	Broth microdilution; MIC	MIC, [*S. aureus*] 2 mg/mL (except for ZnO/S-S-20, C-A-10, or C-A-20, >2) as 0.228–0.632 mg/mL of ZnO NPs, [*C. albicans*] 1 or 2 mg/mL (except for ZnO/S-A-10 or S-S-10 >2) as 0.187–0.632 mg/mL of ZnO NPs	[115]
CuZnO NPs on mesoporous silica SBA-3	Co-condensation method (zinc nitrate)	▪ Highly ordered mesoporous structure with relatively rough surface/not available ▪ NPs: near-spheres/~2 μm (pore size, 3.6274 nm; specific surface area, 829 m^2^/g)	Colony counting; MIC	MIC, [*E. coli*] 25 mg/mL (CuZnO, 0.558 mg/mL), [*S. aureus*] 6.25 mg/mL (CuZnO, 0.139 mg/mL)	[117]
ZnO MPs with TiO_2_ MPs	Sol–gel method (zinc chloride)	▪ ZnO MPs: near-spheres/3.17–10.3 μm ▪ TiO_2_ MP: near-spheres/2.15–37.1 μm	Broth dilution; MIC; photocatalytic incubation under visible light irradiation	ZnO MPs (0.25%) with TiO_2_ MPs (1%): [*E. coli*] 76.8% growth inhibition, [*Streptococcus pyogenes*] 70.2% growth inhibition, [*K. pneumoniae*] 80.8% growth inhibition	[118]
**Combinations of ZnO materials with other biomaterials**					
*Chitosan*					
Chitosan-ZnO NP-loaded gallic acid (C-ZnO@gal) films	Hydrothermal method (zinc sulfate)	▪ ZnO@gal NPs: near-rods/~19.2 nm ▪ C-ZnO@gal films: homogeneous distributed of ZnO@gal NPs on chitosan matrixes (2 g chitosan/70 mg ZnO@gal)/not available (thickness 0.10 mm)	Agar well diffusion	0.5 mg/mL, [*E. coli*] 28 mm zone of inhibition, [*B. subtilis*] 25 mm zone of inhibition	[28]
ZnO NP-containing chitosan coating	Not available (conventional product: 2% *w/v* solution, 10–30 nm)	▪ ZnO NPs: near-spheres/~65 nm ▪ ZnO NPs (1%) in chitosan matrix (2.5%) for coating: polymeric matrixes including uniformly distributed ZnO NPs/not available	Broth dilution; MIC	[*E. coli* O157:H7] ▪ Chitosan (2.5%, *w/v*): 2.5 log (CFU/g) reduction at 4 °C ▪ ZnO NP (1%, *w/v*)-containing chitosan (2.5%, *w/v*): 2.8 log (CFU/g) reduction at 4 °C	[82]
ZnO NP-containing chitosan/gelatin hybrid nanocomposite (nZnO-chitosan/gelatin) films	Green method using *Cassia fistula* fruit extract (zinc nitrate hexahydrate)	▪ ZnO NPs: polyhedrons, quasi-spheres (2% in films), rods (4% in films)/20–40 nm, 500–1000 nm, 200–400 nm ▪ nZnO-chitosan/gelatin films: evenly distributed NPs on smooth, compact, and heterogeneous surface/not available (thickness 86–92 μm)	Disk diffusion	[*E. coli*] ZnO NPs in films: 1%-10.5 mm zone of inhibition, 2%—10.5 mm zone of inhibition, 4%—10.7 mm zone of inhibition [*S. aureus*] not prominent compared to *E. coli*	[91]
3D porous ZnO NP-chitosan/silk/sericin scaffolds for wound dressing	Not available (conventional product: solution, ~35 nm)	Porous microstructures/not available (porosity ~86%; pore size 4–200 μm)	Disk diffusion	1.5 × 1.5 cm^2^, 2% (*w/v*) chitosan, 100 μL and 250 μL of ZnO NPs (40 wt% dispersion), [*E. coli*] 2–4.5 mm zone of growth inhibition, [*S. aureus*] 2.5–5.5 mm zone of growth inhibition	[136]
Chitosan-based ZnO nanocomposites	Co-precipitation (zinc acetate dihydrate)	▪ ZnO NPs: spheres/25–30 nm ▪ Chitosan-based ZnO nanocomposites for biodental meterials: stars/20–25 nm	Disc diffusion	Zone of inhibition (mm), [*K. pneumoniae*] 13 mm (the highest) > [*E. coli*] > [*P. aeruginosa*] > [*B. subtillis*], [*S. aureus*], and [MRSA] 6 mm (the lowest)	[137]
*Hydroxyapatite & alginate*					
Hydroxyapatite-biphasic ZnO NP/MP-embedded alginate beads	Precipitation (zinc nitrate)	▪ ZnO particles: snowflakes/<1 μm ▪ Hydroxyapatite- ZnO-alginate beads: distributed ZnO particles in bead matrixes/not available	Agar diffusion; colony counting; MIC	0.1 mg/mL, [*E. coli*] 56% inhibition, [*P. aeruginosa*] 65% inhibition, [*S. aureus* and *S. epidermidis*] 100% inhibition	[83]
*Polyethylene glycol*					
Tungsten-doped polyethylene glycol-capped ZnO (W-PEG-ZnO) NPs	Electrochemical method (Zn electrodes)	Near-spheres/~40.46 nm	Agar well diffusion	▪ Ampicillin: 100 μg/μL [*E. coli*] 35 mm zone of inhibition, [*B. cereus*] 20 mm zone of inhibition ▪ W-PEG-ZnO NPs: [*E. coli*] 400–600 μg/μL, 5–6 mm zone of inhibition, [*B. cereus*] 300–600 μg/μL, 6–8 mm zone of inhibition	[120]
*K-carrageenan*, *bacterial cellulose*, *propolis extract& curcumin*					
Biopolymer K-carrageenan wrapped ZnO (KC-ZnO) NPs	Precipitation (zinc acetate dehydrate)	KC-ZnO NPs: ovals/97 nm	Disk diffusion; MIC	[MRSA] MIC, 7.50 μg/mL	[27]
Bacterial cellulose-ZnO NP-propolis extract (BC-ZnO-propolis) films	Ultrasound (zinc acetate)	▪ ZnO NPs: quasi-spheres/70–90 nm BC-ZnO- propolis films: homogeneously distributed ZnO NPs on BC substrate surface/not available	Disk diffusion; MIC; photocatalytic incubation (254 nm, 30 min)	MIC, [*E. coli*] >1.89 mg/mL, [*B. subtilis*] 0.44 or <0.44 mg/mL, [*C. albicans*] >0.8, 1.3, 1.89, >1.89 mg/mL	[121]
Curcumin-loaded ZnO (C-ZnO) NPs	Sol–gel method (zinc nitrate hexahydrate)	▪ C-sZnO: spheres/40–100 nm ▪ C-rZnO: rods/length, 600–900 nm (width, <300 nm) ▪ C-jZnO: javelin/300–600 nm (width, <300 nm) ▪ C-spZnO: short petal nanoflower/2–4 μm ▪ C-lpZnO: long petal nanoflower/~500 nm	Well diffusion; colony counting	Zone of inhibition (mm), [*E. coli*] C-sZnO, 8.4; C-rZnO, 10.1; C-jZnO, 11.1; C-spZnO, 8.1; C-lpZnO, 9.8; C, 7.4 [*S. epidermis*] C-sZnO, 19.1; C-rZnO, 17.2; C-jZnO, 19.4; C-spZnO, 16.4; C-lpZnO, 20.1; C, 8.2 [*S. aureus*] C-sZnO, 15.4; C-rZnO, 16.6; C-jZnO, 13.4; C-spZnO, 15.2; C-lpZnO, 17.0; C, 8.1 [*B. cereus*] C-sZnO, 17.4; C-rZnO, 18.7; C-jZnO, 18.7; C-spZnO, 14.2; C-lpZnO, 14.2; C, 8.4	[124]
*Graphene, graphene oxide, & reduced graphene oxide*					
Graphene/ZnO nanocomposite films	Ion exchange process (zinc acetate)	▪ ZnO NPs: near-spheres/20–40 nm ▪ Graphene/ZnO nanocomposite films: ZnO NP-distributed graphene sheet/not available	Microdilution; MIC	[*Streptococcus mutans*] MIC, 125 μg/mL	[84]
Graphene/ZnO nanocomposite with curcumin (C-ZnO)	Ion exchange process (zinc acetate dihydrate)	▪ ZnO NPs: spheres/35 nm ▪ Graphene/ZnO: homogenous distribution of ZnO NPs on graphene sheet/not available	Agar diffusion; colony counting; microdilution; MIC	MIC, ▪ Curcumin: 125 μg/mL ▪ Graphene/ZnO: [MRSA ATCC 43300] 125 μg/mL, [MRSA ATCC BAA-1708] 250 μg/mL ▪ Graphene/C-ZnO: [MRSA ATCC 43300] 31.25, [MRSA ATCC BAA-1708] 62.5 μg/mL (~64% inhibition of in vivo MRSA topical dermatitis infection)	[85]
Graphene oxide (GO)/ZnO nanocomposite for wound care	Co-precipitation (zinc nitrate)	▪ GO: smooth and wrinkled surface layers/not available▪ GO/ZnO nanocomposite: well incorporated and distributed ZnO NPs (0.1–0.4 M) on GO sheets forming agglomerates/not available	Disk diffusion, colony counting; dark (D) and visible light-irradiated (L) conditions	Zone of growth inhibition (mm), ▪ ZnO NPs (0.4 M) on GO sheets, 100 μg/mL [*E. coli*] GO-D, 11 mm; L, 11.5 mm; GO/ZnO (0.4 M)-D, 11 mm; L, 13 mm [*P. aeruginosa*] GO-D, 10 mm; L, 10.5 mm; GO/ZnO (0.4 M)-D, 10 mm; L, 13 mm [*S. typhi*] GO-D, 10.5 mm; L, 9 mm; GO/ZnO (0.4 M)-D, 11 mm; L, 11.5 mm [*S. flexneri*] GO-D, 8 mm; L, 10.6 mm; GO/ZnO (0.4 M)-D, 12 mm; L, 12.5 mm	[86]
GO/ZnO composites	Ion exchange process (zinc acetate dihydrate)	▪ ZnO NPs: rods/4 nm ▪ GO/ZnO composites: homogeneously anchored ZnO NPs onto GO sheets/not available	Agar disk diffusion; MIC	[*E. coli*] MIC, 2 μg	[87]
Reduced graphene oxide (rGO)/ZnO films	Sol–gel synthesis (zinc acetate dihydrate)	▪ ZnO NPs: spheres/~100–300 nm ▪ rGO/ZnO films: homogenous distribution of ZnO NPs on rGO sheet/not available (roughness, 159 nm)	Serial dilution; colony counting; MIC; photocatalytic incubation (UV at 365 nm)	[*S. aureus*] 1 wt% rGO >99% (>2-log) reduction	[88]
*Cotton fabric*					
ZnO MPs-loaded chitosan-coated cotton fabrics	Precipitation (zinc chloride)	Uniformly distributed dense microstructure of rods/not available	Disk diffusion	[*E. coli*] 2.5 cm zone of growth inhibition (ZnCl_2_ 4%, chitosan 1–2%/1 g cotton fabric)	[126]
Cotton-ZnO NP composites (C-nZnO)	Precipitation (zinc chloride)	▪ ZnO NPs: quasi-spheres/27 nm ▪ C-nZnO composites (8 types): thick condense layers of ZnO NPs on cotton surfaces/not available	Disk diffusion; colony counting	nZnO amounts in C-nZnO: 2.2, 1.7, 4.9, 4.3, 11.1, 7.8, 22.2, and 16.7 wt% 9 mm in diameter, [*E. coli*] 97–100% growth reduction, [*S. aureus*] 96–98% growth reduction	[127]
ZnO NP-coated fabric	Green method using starch (zinc nitrate)	▪ ZnO NPs: spheres/200 nm ▪ ZnO NP-coated fabric: evenly distributed ZnO NPs on fabric surface/not available	Colony counting	4.8 cm in diameter of fabric, [*E. coli*] 80% reduction [*S. aureus*] 99.99% reduction	[129]
**ZnO quantum dots (QDs)**					
Different nanostructure-based ZnO QDs (ZnO QD-1–ZnO QD-14)	Sol–gel method (zinc acetate dihydrate)	Nanorods, nanotubes, nanospheres, nanowhiskers, nanoflowers/not available	Agar well diffusion; agar dilution; MIC	MIC, [*E. coli*] ZnO QD-1: 25 mg/mL, [*Enterobacter aerogenes*] ZnO QD-4 and ZnO QD-6: 25 mg/mL, [*K. pneumonia*] ZnO QD-3 and ZnO QD-5: 12.5 mg/mL, [*P. aeruginosa*] ZnO QD-3 and ZnO QD-7: 12.5 mg/mL, [*Bacillus anthracis*] ZnO QD-2, ZnO QD-3 and ZnO QD-8: 6.25 mg/mL, [*S. aureus*] ZnO QD-8: 6.25 mg/mL, [*L. monocytogenes*] ZnO QD-6 and ZnO QD-7: 50 mg/mL, [*E. faecalis*] ZnO QD-2 and ZnO QD-7: 25 mg/mL, [*B. cereus*] ZnO QD-3 and ZnO QD-5: 12.5 mg/mL, [*S. epidermidis*] ZnO QD-8: 1.5 mg/mL	[89]
ZnO QDs	Green method using *Eclipta alba* leaf extract (zinc acetate dihydrate)	Spheres/~6 nm	Agar diffusion	[*E. coli*] 15.69 mm zone of inhibition (1.6-fold increase compared to bulk zinc acetate at 5 mM)	[90]
Antimicrobial peptide-based ZnO QDs containing vancomycin and methicillin (Van@ZnO-BSA-PEP-MPA; Met@ZnO-BSA-PEP-MPA)	Precipitation (zinc acetate)	ZnO@BSA-PEP-MPA: spheres/104 nm	Broth dilution; MIC; in vivo diagnostics-4 × 10^8^ CFU *S. aureus* for infection and Van@ZnO-BSA-PEP-MPA at 5.0 mg/kg for theranostics	MIC, ▪ ZnO@BSA-PEP-MPA: no inhibition (nanoprobe) ▪ Van@ZnO-BSA-PEP- MPA: [*S. aureus*] 2.0 μg/mL, [*B. subtilis*] 1.0 μg/mL (*in vivo* diagnostics: no changes in activity and body weight, 6 × 10^4^ log (CFU/g) growth inhibition) ▪ Met@ZnO-BSA-PEP- MPA: [MRSA] 64 μg/mL	[141]
Polyvinylpyrrolidone-capped ZnO (PVP-ZnO) QDs	Precipitation (zinc acetate hydrate)	▪ ZnO QDs: spheres/~5 nm ▪ PVP-ZnO QDs: highly crystalline spheres/~4 nm (smaller than ZnO QDs)	Agar diffusion; colony counting	▪ ZnO QDs (1.12 mg/mL): [*S. Enteritidis*] 63.9% growth inhibition, [*L. monocytogenes*] 80.6% growth inhibition ▪ PVP-ZnO QDs (40 mg/mL): [*E. coli O157:H7*] 66.7% growth inhibition, [*L. monocytogenes*] 58.9% growth inhibition	[142]

## Abbreviations

BC	Bacterial cellulose
BSA	Bovine serum albumin
CFU	Colony forming unit
CFX	Ceftriaxone
CFZ	Ceftazidime
C-jZnO	Curcumin-loaded ZnO javelin
C-lpZnO	Curcumin-loaded long petal zno nanoflower
C-nZnO	Cotton-ZnO nanoparticle nanocomposite
C-rZnO	Curcumin-loaded ZnO rod
C-spZnO	Curcumin-loaded short petal zno nanoflower
C-sZnO	Curcumin-loaded ZnO sphere
C-ZnO	Curcumin-loaded ZnO
C-ZnO@gal	Chitosan-ZnO nanoparticle-loaded gallic acid
D	Dark
GO	Graphene oxide
GTM	Gentamicin
HA-ZnO-Alg	Hydroxyapatite-biphasic ZnO nanoparticle/microparticle-embedded alginate
HSV-2	Herpes simplex virus type-2
KC	K-carrageenan
L	Light
Met	Methicillin
MIC	Minimum inhibitory concentration
MPs	Microparticles
MRSA	Methicillin-resistant staphylococcus aureus
nZnO-chitosan/gelatin	ZnO NP-containing chitosan/gelatin hybrid nanocomposite
PE	Polyethylene
PVP	Polyvinylpyrrolidone
QDs	Quantum dots
rGO	Reduced graphene oxide
UV	Ultraviolet
Van	Vancomycin
W-PEG-ZnO	Tungsten-doped polyethylene glycol-capped ZnO
